# Environmental stress linked to consumption of maternally derived carotenoids in brown trout embryos (*Salmo trutta*)

**DOI:** 10.1002/ece3.3076

**Published:** 2017-06-02

**Authors:** Laetitia G. E. Wilkins, Lucas Marques da Cunha, Gaëtan Glauser, Armelle Vallat, Claus Wedekind

**Affiliations:** ^1^ Department of Ecology and Evolution Biophore, University of Lausanne Lausanne Switzerland; ^2^ Department of Environmental Sciences Policy & Management University of California Berkeley CA USA; ^3^ Neuchâtel Platform of Analytical Chemistry University of Neuchâtel Neuchâtel Switzerland

**Keywords:** astaxanthin, brown trout, embryo survival, lutein, maternal effects, Salmonidae, Zeaxanthin

## Abstract

The yellow, orange, or red colors of salmonid eggs are due to maternally derived carotenoids whose functions are not sufficiently understood yet. Here, we studied the significance of naturally acquired carotenoids as maternal environmental effects during embryo development in brown trout (*Salmo trutta*). We collected eggs from wild females, quantified their egg carotenoid content, fertilized them in vitro in full‐factorial breeding blocks to separate maternal from paternal effects, and raised 3,278 embryos singly at various stress conditions until hatching. We found significant sire effects that revealed additive genetic variance for embryo survival and hatching time. Dam effects were 5.4 times larger than these sire effects, indicating that maternal environmental effects play an important role in determining embryo stress tolerance. Of the eight pigment molecules that we targeted, only astaxanthin, zeaxanthin (that both affected egg redness), and lutein were detected above our confidence thresholds. No strong link could be observed between carotenoid content in unfertilized eggs and embryo mortality or hatching timing. However, the consumption of carotenoids during our stress treatment was negatively correlated to embryo survival among sib groups and explained about 14% of the maternal environmental variance. We conclude that maternally derived carotenoids play a role in the ability of embryos to cope with environmental stress, but that the initial susceptibility to the organic pollution was mainly determined by other factors.

## 1 INTRODUCTION

The phenotype of an embryo is determined by its genes, the environment, and the support it receives from its parents. In egg‐laying species with no parental care, parental effects are confined to maternal investments into eggs. These maternal investments may reveal variation in maternal nutritional state, health and vigor, or different types of life‐history trade‐offs (Nordeide, Kekalainen, Janhunen, & Kortet, 2013). In the case of the brown trout (*Salmo trutta*) and other salmonid fishes, there is typically much within‐population variation in egg size and egg color. Within‐clutch variation in these traits is small compared to the strong differences among females who can adjust their optimal egg and clutch sizes between breeding seasons depending on environmental conditions and maternal phenotype (Hendry & Day, 2003; Kinnison, Unwin, Hendry, & Quinn, 2001; Parker & Begon, 1986). Egg size is usually correlated to female size and affects offspring growth (Einum, 2003; Einum & Fleming, 1999) and resistance to oxygen stress (Einum, Hendry, & Fleming, 2002). The mean egg color per female can vary from a pale yellow, to brightly orange or intense red. Such colors are due to carotenoids (Goodwin, 1984) that can be allocated to eggs in different combinations and concentrations.

Carotenoids are lipid‐soluble hydrocarbon pigments that are synthesized by plants and some microbes and that animals need to obtain through their diet (Goodwin, 1984). These pigment molecules can play numerous physiological roles (Blount, Houston, & Møller, 2000) that affect, for example, the respiratory efficiency of an organism (Hill & Johnson, 2012; Tomášek et al., 2016), the antioxidant activity against the damage caused by free radicals (Krinsky, 1991), or the immune response where some carotenoids have been shown to support the production of antibodies and the proliferation of immune cells (McGraw & Ardia, 2003; Peters, 2007). Moreover, carotenoids also represent precursors of retinoids that are not only involved in immune functioning but also in vision and the healthy development of embryos (Blomhoff & Blomhoff, 2006; Stephensen, 2001). Hence, carotenoids can increase the fitness of both the mother and her offspring (Møller et al., 2000).

In salmonid fishes, carotenoids can be stored in muscles, skin, or eggs, and get mobilized to skin and eggs during the breeding season (Garner, Neff, & Bernards, 2010). Despite the general expectancy that more intensely colored eggs may therefore be of higher quality (e.g., Palace & Werner, 2006), studies on hatchery‐reared eggs of wild or domestic origin do not always support such an assumption. For example, Tyndale, Letcher, Heath, and Heath (2008) found in Chinook salmon (*Oncorhynchus tshawytscha*) that egg carotenoid content was positively correlated to incubation survival in some wild populations, but not in others. In one domestic strain, the correlations between egg carotenoid content and incubation survival were even negative and close to statistical significance. Supplementary feeding of astaxanthin typically increases its content in the eggs (McCallum, Cheng, & March, 1987; Sawanboonchun, Roy, Robertson, & Bell, 2008; but see also Brown, Leonard, McGraw, & Clotfelter, 2014). Increased astaxanthin content in eggs may then be positively correlated to fertilization and hatching rates in some artificial environments, as found, for example, in rainbow trout (*O. mykiss*; Ahmadi, Bazyar, Safi, Ytrestoyl, & Bjerkeng, 2006), Atlantic cod (*Gadus morhua*; Sawanboonchun et al., 2008), or carp (*Cyprinus carpio;* Sowmya & Sachindra, 2015). However, there seem to be strong dose dependencies. High levels of certain carotenoids are sometimes equally beneficial (Anbazahan et al., 2014) or even less beneficial (Amar, Kiron, Akutsu, Satoh, & Watanabe, 2012; Brown, Cahn, Choi, & Clotfelter, 2016; Kolluru et al., 2006; Vinkler & Albrecht, 2010) than intermediate ones.

In the wild, salmonid embryos develop over a period of several weeks as free‐living and nonmobile organisms in their aquatic environment and are hence very exposed to various environmental stressors. Maternally derived carotenoids may then be important due to their likely beneficial effects on stress tolerance (Blomhoff & Blomhoff, 2006; Pechinskii & Kuregyan, 2014). However, the role that carotenoids play here is not sufficiently understood (e.g., Hartley & Kennedy, 2004; Tomášek et al., 2016). Do they help prevent stress or do they help fight the effects of stress? In the first case, we would expect positive correlations between carotenoid contents and indicators of stress (e.g., embryo survival) and in the second case correlations between the amount of stress that a group of embryos experiences and their consumption of carotenoids. Variation in embryo stress could first depend on egg characteristics that are not linked to carotenoids; that is, on other maternal environmental effects (e.g., egg size, characteristics of the egg membrane, presence and concentrations of other egg components; Løvoll et al., 2006), and on embryo genetics, as found in brown trout (Clark, Stelkens, & Wedekind, 2013; Jacob, Evanno, von Siebenthal, Grossen, & Wedekind, 2010; Wedekind, Jacob, Evanno, Nusslé, & Müller, 2008) and other salmonids (e.g., Aykanat, Heath, Dixon, & Heath, 2012; Pitcher & Neff, 2006; Wedekind et al., 2004). The nature of correlations between carotenoid content and consumption to embryo survival indicates the importance of carotenoids at different lines of defense. A positive correlation between initial carotenoid content and embryo viability would indicate that carotenoids help prevent stress, whereas a positive correlation between carotenoid consumption and stress indicators would suggest that some maternal sib groups are more susceptible to stress than others.

Here, we sampled brown trout from the wild, identified and quantified the carotenoids that females allocated to their eggs, and linked them to egg color and female phenotype, including skin coloration. We then used block‐wise full‐factorial in vitro fertilizations to experimentally separate dam from sire effects on embryo survival and time until hatching. We reared the resulting embryos singly until hatching at different levels of organic pollution that create stressful environments for the embryos (Jacob et al., 2010; Wedekind, Gessner, Vazquez, Maerki, & Steiner, 2010). Adding nutrient broth to brown trout embryos has been shown to increase embryo mortality, change the egg‐associated bacterial community composition, and cause a transition in their functional gene pathways (Wilkins, Fumagalli, & Wedekind, 2016). Increased mortality rates in nutrient broth‐treated embryos could even be linked to a lower allelic diversity in their immune system (major histocompatibility complex; Jacob et al., 2010). This experimental setup allowed us to quantify the variance components of embryo viability (additive genetic variance and maternal environmental variance; i.e., all parental effects) under stress and benign conditions while controlling for possibly confounding factors. We performed a second quantification of carotenoids in embryos 14 days after the stress treatment to quantify carotenoid consumption per maternal sib groups and to relate it to the mean survival and hatching timing of the 126 half‐sib groups that we monitored in this study. The main aims of this study are to (1) provide information about naturally allocated carotenoids (and their within‐population variation) in eggs and embryos of wild brown trout, (2) test for correlations between egg carotenoid content to egg and female phenotypes, (3) test whether carotenoid content and consumption predict embryo viability (survival and hatching time) under environmental stress, (4) quantify the relative importance of additive genetic variance and maternal environmental variance, and (5) estimate how much of the maternal environmental variance in embryo viability is explained by egg carotenoids.

## 2 MATERIALS AND METHODS

### 2.1 Sample acquisition

Two tributaries of the river Aare were sampled for the experiments: the river Müsche (7°30′30,21″/46°50′43,30) and the river Kiese (7°37′11,27/46°50′55,85). Adult brown trout (*Salmo trutta* morpha *fario*) were caught with electrofishing from their spawning grounds and kept at the *Fischereistützpunkt Reutigen* until they could be stripped of their gametes. These gametes were subsequently used for full‐factorial in vitro fertilizations following the methods described in Jacob et al. (2010). Five unfertilized eggs per female were used to measure their carotenoid content. Previous laboratory tests (unpublished preliminary analyses) had shown that it was necessary to pool five eggs in order to reach the detection limit threshold of ultra‐high‐pressure liquid chromatography‐diode array detection.

### 2.2 Extraction and quantification of carotenoids

All five eggs were simultaneously homogenized in 1 ml ethyl acetate (pure solvent) with five glass beads (2 mm, VWR International, Radnor, USA) in a mixer mill (MM300; Retsch, Düsseldorf, Germany) for 2 × 1 min at a frequency of 30 Hz. The homogenate was centrifuged at full speed (i.e., 16,162***g***) for 2.5 min. The supernatant was kept on ice, and the pellet went through a second step of bead beating with the residual glass beads and 0.5 ml of fresh ethyl acetate. After centrifugation at full speed for 2.5 min, both supernatants were combined and 1 ml of water was added. After sonication for 30 s, the mix was centrifuged at full speed for 1 min. The upper phase was kept on ice again while the lower phase was sonicated again for 30 s in the presence of 1 ml of fresh ethyl acetate. After centrifugation at full speed for 1 min, both upper phases were combined and dried in a centrifugal evaporator (Centrivap, Labconco, Kansas City, USA) for 70 min with the centrifuge kept at 35°C. The residue was dissolved in 150 μl of tetrahydrofuran (THF; pure solvent). The extract was always kept in the dark (using aluminum foils). We quantified carotenoid content as the amount of extracted carotenoids from five pooled unfertilized eggs irrespectively of their size that was then dissolved in THF (μg of carotenoids/ml of solvent).

Carotenoid contents of eggs were measured using ultra‐high‐pressure liquid chromatography (UHPLC) coupled to photodiode array (PDA) detection. The injection volume per sample was 2.5 μl. The separation was carried out on an Acquity UPLC^™^ (Waters, Baden, Switzerland) using an Acquity BEH C18 column (2.1 × 50 mm, 1.7 μm, Waters) under the following conditions: mobile phase A = water, mobile phase B = acetonitrile; 75–87.5% B in 5 min, 87.5–100% B in 1.0 min, holding at 100% B for 2.0 min, re‐equilibration at 75% B for 1.5 min. The flow rate was set to 0.750 ml/min. Autosampler and column temperatures were kept at 15 and 45°C, respectively. UV spectra were acquired over the range 210–600 nm at a frequency of 10 Hz. The extracted trace at 450 nm was used for quantification. The following pigment molecules were targeted: asthaxanthin, canthaxanthin, beta‐carotene, beta‐cryptoxanthin, lutein, all‐trans‐retinol, retinyl‐palmitate, and zeaxanthin. The carotenoids astaxanthin (retention time “RT” 1.32 min), lutein (RT 1.98 min), and zeaxanthin (RT 1.88 min) could be detected above the confidence limit of the UHPLC method and were quantified by external calibration using reference standards purchased from certified distributors. Traces of some of the remaining carotenoids could be detected but were not used because sensitivity and selectivity were too low. No carotenoid contents could be determined for the eggs of one dam because the respective samples were accidentally destroyed (Table S1). The repeatability of our method was validated by Rottet et al. (2016) and Spicher, Glauser, and Kessler (2016).

### 2.3 Female phenotypes

Directly after stripping, each female was photographed. Included in each photograph was a size and color standard against which all photographs could be calibrated. These photographs were used later to determine female body length and coloration in ImageJ v.1.49u (Schneider, Rasband, & Eliceiri, 2012). Prior to the color analyses, the white balance of the photos was corrected using the color scale as a reference. Redness of the mothers was measured as (1) the proportion of red area relative to the total body area of the individual's left side and (2) relative redness of the red spots on a fish. Both measures were included because preliminary explorations of our dataset revealed that the two measures were not correlated (unpublished results). We measured both redness estimations in the laboratory color space, a color‐opponent space in which “L” is the lightness, and “a” and “b” are the color‐opponent dimensions. The area of all red spots present on the left body side was measured in pixels. Then, this area was divided by the total body area on the same body side. Redness of the red spots was quantified by measuring the “a” component of laboratory. In order to standardize these measurements with the lightness, we divided “a” by “L.” Relative redness was estimated by dividing the mean redness of the red spots by the mean redness of the color scale present in each picture. Darkness of the skin of the mothers was measured as mean gray values (i.e., the mean of the color channels RGB) extracted from the whole left body side of females.

### 2.4 Egg phenotypes

Five unfertilized eggs per female were photographed on a microscope slide under standardized light conditions and at a standard distance, including a size and a color standard in each picture. These pictures were later used to measure egg redness in ImageJ, analogously to the measurements of female redness above. Egg sizes were calculated by measuring egg areas individually for all five eggs on the same picture with the lasso tool and multiplying this measure by 2/3 of individual egg diameters to get egg volumes. The mean volume of five eggs was used for statistical analyses. Variances in egg redness and sizes were analyzed with an ANOVA. Correlations among different carotenoid contents and their changes were estimated using Pearson's product moment correlations (*r*) in order to find out whether they could be used as independent predictors in our models describing embryo viability (survival and hatching time). These correlations also served as a way to validate our extraction method as each sample was extracted independently. The relationship between egg redness and its carotenoid contents was analyzed in a multiple regression including all carotenoids.

### 2.5 Experimental protocol of trout embryos

Twelve females from the river Kiese were crossed full factorially with eight males from the river Kiese (96 families), and six females from the river Müsche were crossed full factorially with five males from the river Müsche (30 families), resulting in two breeding blocks and in total 126 half‐sib families. All fertilized eggs were distributed singly to 24‐well plates (Falcon, BD Biosciences, Allschwil, Switzerland) filled with 2 ml water/well that had been standardized according to OECD guidelines (OECD, 1992). The eggs were then incubated at 6.5°C in a climate chamber. Once embryos had reached the late‐eyed developmental stage (45 days after fertilization), nonfertilized eggs and dead embryos were removed, a subset of embryos from the river Müsche used for another experiment (unpublished data), and the remaining embryos (*N* = 3,648) re‐distributed singly to new 24‐well plates where they were immediately exposed to one of two different concentrations (1:1,000 or 1:500) of nutrient broth (“NB”: 3 g meat extract and 5 g bactopeptone per 1 L distilled H_2_O), or sham treated with distilled H_2_O.

Fourteen days after treatment (59 days after fertilization) when new microbial communities had been established on the eggs (Wedekind et al., 2010), five embryos per female were randomly sampled from the highest stress treatment group (NB 1:500) and stored at −80°C (without storage buffer) for a second quantification of astaxanthin, lutein, zeaxanthin. At the same day, five other embryos per family and treatment of a subset of the river Kiese breeding block were sampled for a parallel study on bacterial communities on eggs. This subset included all half‐sib groups from eight dams crossed full factorially with seven sires (Dams 1–8 and Sires 1–7; Table S1). These samples were used for the characterization of egg‐associated microbiotas based on high‐throughput sequencing, and for comparisons of bacterial communities to the survival of the corresponding full‐sibs. See Wilkins et al. (2016) for the corresponding analysis of the influence of treatment, dam, sire, and bacterial diversity on embryo survival, hatching timing, and bacterial diversity within these 56 half‐sib families. Here, we include these survival data into the larger sample (126 half‐sib families in total) in order to test for correlations to carotenoids.

**Figure 1:**
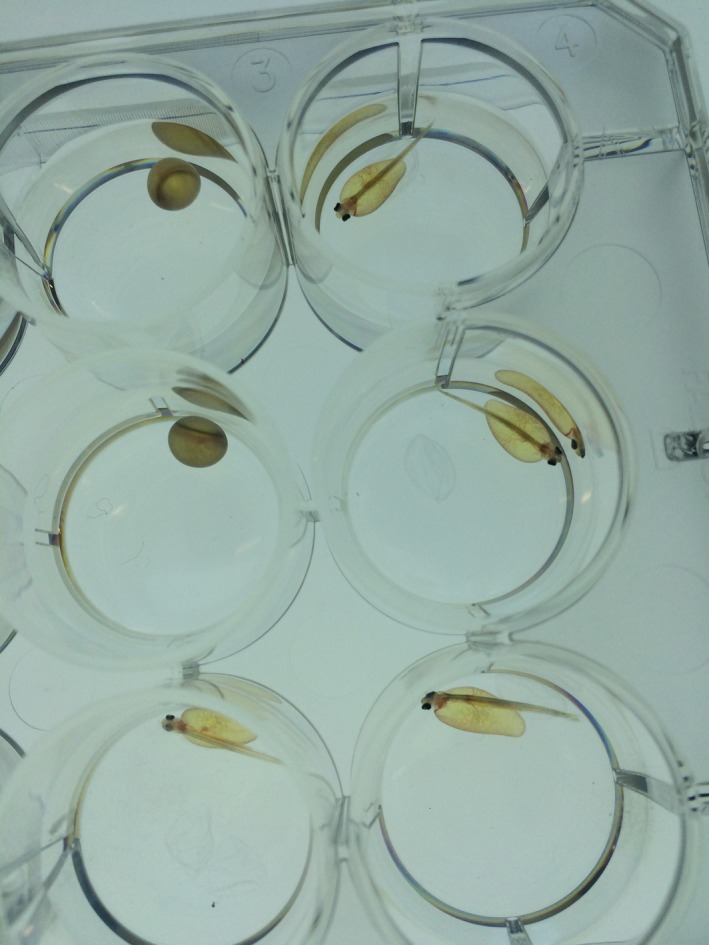
Brown trout embryos and freshly hatched larvae in their individual wells as used in this study

After the sampling at day 59 after fertilization, all remaining embryos (*N*_total_ = 3,278; ranging from 15 to 45 replicates per half‐sib group and treatment) were transferred to new 24‐well plates with 2 ml of fresh standardized water (if alive; total mortality includes five embryos that died during incubation). They were then daily monitored until hatching (Figure 1) to record individual embryo mortality and time until hatching.

### 2.6 Viability analysis

Survival in response to treatment was analyzed as in Bolker et al. (2009): Embryo survival was entered as a binary response variable (dead before hatching or hatched) in generalized linear mixed models (GLMM) treating each embryo as independent replicate. Treatment and carotenoid contents (astaxanthin and zeaxanthin) in five pooled unfertilized eggs) were entered as fixed effects. To avoid collinearity problems, we did not include lutein in the models as it turned out to be significantly correlated to zeaxanthin (see results section). An analogous model with astaxanthin and lutein is shown in the Supplementary Material. Dam, sire, population, and all possible two‐way interactions were entered as random effects. Starting with a reference model, one main effect or one interaction effect at a time was removed or added. The goodness of fit of the different models is given both by the logarithm of the approximated likelihood and by the Akaike's information criterion (AIC). Models were compared with likelihood ratio tests (LRT), with the difference in number of free parameters in the two models as degrees of freedom. The difference of AICs between two models (δAIC) was used to compare the quality of fit. The lme4 package v.1.1.7 for logistic mixed effect model analyses was used (Bates, Maechler, Bolker, & Walker, 2015). To test whether redness of the eggs would explain a significant part of the variance in offspring mortality, an alternative reference model was fitted with carotenoid content replaced by redness of the eggs. Analogous linear mixed models were used to analyze the variances in time until hatching.

The effects of dam, sire, and carotenoid contents were also investigated within treatments. Because carotenoid contents were measured again 14 days after treatment within the higher stress treatment (NB 1:500), the difference of carotenoid contents before fertilization and 14 days after treatment could be analyzed in analogous models for this treatment group. However, as all three carotenoid changes showed a strong correlation (see results section), we ran three individual models only including one carotenoid type at a time. We tested whether females that show greater changes in carotenoid contents also produce offspring with more mortality in the high nutrient broth treatment. The relationship between mean carotenoid changes and hatching times per female were investigated with Pearson's product moment correlations.

Besides random dam effects we also investigated with corresponding GLMMs whether embryo survival and hatching times under the different stress conditions could be predicted by other female characteristics; that is, other maternal environmental effects. Here, we included treatment as a fixed effect and sire and dam as random effects in the reference model. We tested the following female traits individually: origin (river Kiese or river Müsche) as a random effect, and size (mm), weight (g), red coloration (proportion of red area relative to the total body area and its relative redness), darkness of the skin (mean gray values), or mean egg size (mm^3^) as fixed effects. Their interaction with treatment was also investigated.

### 2.7 Variance components for parental effects on embryo viability

Variance components for survival and hatching time were extracted within treatments from mixed models using REML. Only astaxanthin measures were entered as a fixed effect to avoid collinearity problems, while dam and sire were entered as random effects. Additive genetic variance (*V*_A_) was estimated as four times the sire variance (*V*_SIRE_) assuming that epistasis and dominance effects are negligible (Lynch & Walsh, 1998). For dam effects, we calculated two variance components: the total maternal variance (*V*_DAM_) and maternal environmental effects (*V*_MENV_). The latter represents the part of *V*_DAM_ that cannot be explained by additive genetic effects, and it is calculated by subtracting *V*_SIRE_ from *V*_DAM_. Variance components for the fixed effect of astaxanthin were directly extracted from the GLMMs according to Nakagawa and Schielzeth (2013). Standard deviations of variance components were estimated using a bootstrap approach where 1,000 datasets were produced by reshuffling the survival data separately for each population and treatment, analogous to Pompini, Clark, and Wedekind (2013). All statistical analyses were performed in R v.3.1.3 (R Development Core Team 2015) and JMP® 11.2.0 (SAS Institute Inc.).

## 3 RESULTS

### 3.1 Egg phenotypes

**Figure 2:**
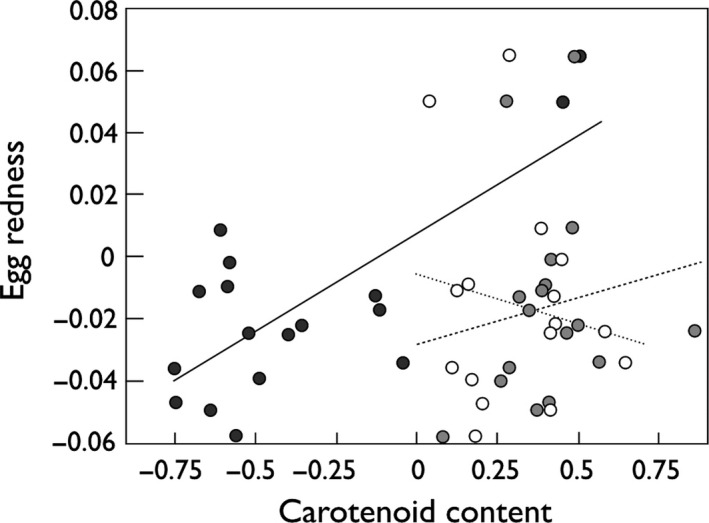
Redness of unfertilized eggs versus their carotenoid content. Correlation to astaxanthin: black symbols and nonhatched regression line; zeaxanthin: gray symbols and hatched regression line, and lutein: open symbols and dotted regression line. Carotenoid content in μg/ml and log_10_‐transformed. See text for statistics

Mean carotenoid contents in five unfertilized eggs were as follows: astaxanthin = 0.70 μg/ml (95% CI: 0.23–1.16), lutein = 2.21 μg/ml (1.74–2.67), and zeaxanthin = 2.75 μg/ml (2.09–3.42). These means correspond to astaxanthin = 1,168.2 nmol/L, lutein = 4,110.3 nmol/L, and zeaxanthin = 4,840.4 nmol/L (Table S1). The red color of the unfertilized eggs was correlated to carotenoid contents (Figure 2; multiple regression with log_10_‐transformed carotenoid contents, *N* = 17, effects of astaxanthin: *t *=* *4.9, *p *<* *.001; zeaxanthin: *t *=* *2.3, *p *=* *.04; lutein: *t *=* *−2.5, *p *=* *.03). Variances in egg redness and egg sizes were significantly greater between females than within females (Table S2). Mean astaxanthin content per dam was not correlated to mean lutein or zeaxanthin content (*r* always <.03, *N* = 17, *p* always >.9), while mean lutein and zeaxanthin contents were correlated (*r* = .68, *p *=* *.003).

### 3.2 Viability of embryos until hatching

No over‐dispersion was found, and dam x sire interaction effects did not have a significant influence on survival in any model and could therefore be excluded from the reference models (Table 1). As expected, the addition of nutrient broth to the wells significantly reduced embryo survival, and mortality increased with elevated nutrient broth concentrations (Table 1; control = 6.3%, NB 1:1,000 = 13.3%, NB 1:500 = 24.2%). Embryo survival varied also among sires and dams (Table 1); that is, there was additive genetic variance for survival. The δAIC of the dam effect was 5.4 times larger than the δAIC of the sire effect (Table 1), suggesting significant maternal environmental effects on embryo survival. There were significant interactions of treatment with dam effects and astaxanthin content (Table 1). Zeaxanthin contents in unfertilized eggs did not affect embryo survival in response to treatment (Table 1). The results did not change when zeaxanthin was exchanged with lutein in the models (Table S3).

**Table 1:** Likelihood ratio tests of logistic mixed model regressions on trout embryo survival

Model	Effect tested	Model parameters		AIC	ln L	Likelihood ratio tests		
		Fixed	Random			δAIC	**χ** ^**2**^	*p*
**Reference model**		**T,A,Z**	**D,S**	**2,221.1**	−**1,103.5**			
Model 1	T	A,Z	D,S	2,377.2	−1,183.6	156.1	160.2	<.0001
Model 2	A	T,Z	D,S	2,219.2	−1,103.6	1.9	0.13	.72
Model 3	Z	T,A	D,S	2,219.2	−1,103.6	1.9	0.13	.71
Model 4	D	T,A,Z	S	2,348.6	−1,168.3	127.5	129.6	<.0001
Model 5	S	T,A,Z	D	2,244.9	−1,116.4	23.8	25.8	<.001
Model 6	P	T,A,Z	D,S,P	2,222.8	−1,103.4	1.7	0.28	.6
**Redness reference**		**T**	**D,S**	**2,317.1**	−**1,153.5**			
Model 7	R	T, R	D,S	2,319.1	−1,153.5	2	0.02	.86
Interaction models								
Model 8	DxS	T,A,Z	D,S	2,383.6	−1,092.1	162.5	19	1
Model 9	TxD	T,A,Z	S	2,208.6	−1,092.3	12.6	22.5	.0004
Model 10	TxS	T,A,Z	D	2,228.4	−1,102.2	7.3	2.6	.75
Model 11	TxA	T,Z	D,S	2,209.3	−1,097.7	11.8	11.7	.002
Model 12	TxZ	T,A	D,S	2,221.5	−1,101.8	0.4	3.5	.17

Different logistic mixed effects models were compared to a reference (in bold) to test if the effects of treatment (T), astaxanthin (A), zeaxanthin (Z), dam (D), sire (S), population (P), redness of the eggs (R), and the interactions dam x sire (DxS), treatment x dam (TxD), treatment x sire (TxS), treatment x astaxanthin (TxA), and treatment x zeaxanthin (TxZ) explain a significant part of the variance in embryo survival (carotenoid contents were measured in five unfertilized eggs). Significant effects are highlighted.

When testing for main effects within treatment groups, dam and sire effects were always significant (Table 2ca‐). Astaxanthin content in unfertilized eggs seemed to correlate with embryo survival at the highest concentration of nutrient broth (Table 2c). However, the corresponding *p*‐value that the statistical model provides is two‐tailed, while we had strong a priori expectancies about the direction of this correlation. Here, the correlation was in the unexpected direction; that is, higher astaxanthin content in unfertilized eggs would lead to increased vulnerability to stress. We therefore adjusted the alpha value to perform directed testing for effects in the unexpected direction (i.e., α = 0.01; Rice & Gaines, 1994), which is below the observed *p*‐value in Table 2c. We therefore conclude that we found no significant correlations between carotenoid contents in unfertilized eggs and embryo tolerance to the stress we inflicted. These results did not change when we exchanged zeaxanthin with lutein in the models (Table S4).

**Table 2:** Likelihood ratio tests of logistic mixed model regressions on trout embryo survival within treatments

Model	Effect tested	Model parameters		AIC	ln L	Likelihood ratio tests		
		Fixed	Random			δAIC	**χ** ^**2**^	*p*
a) Controls								
**Reference model**		**A,Z**	**D,S**	**386.2**	**−188.1**			
Model 1	A	Z	D,S	387.6	−189.8	1.4	3.1	.07
Model 3	Z	A	D,S	387.6	−189.8	1.4	3.4	.06
Model 4	D	A,Z	S	426.1	−209.1	39.9	41.8	<.0001
Model 5	S	A,Z	D	394.2	−193.1	8	9.9	.001
**Redness reference**		**1**	**D,S**	**422.6**	**−207.3**			
Model 6	R	R	D,S	420.7	−207.3	1.9	0.09	.8
b) NB 1:1,000								
**Reference model**		**A,Z**	**D,S**	**741.8**	**−365.9**			
Model 1	A	Z	D,S	740.4	−366.2	1.4	0.6	.44
Model 3	Z	A	D,S	741.9	−366.9	0.1	2.1	.14
Model 4	D	A,Z	S	791.8	−391.9	50	51.9	<.0001
Model 5	S	A,Z	D	746.2	−369.1	4.4	6.4	.01
**Redness reference**		**1**	**D,S**	**753.9**	**−372.9**			
Model 6	R	R	D,S	752.2	−373.1	1.7	0.4	.5
c) NB 1:500								
**Reference model**		**A,Z**	**D,S**	**1,126**	**−557.9**			
Model 1	A	Z	D,S	1,128.9	−560.4	2.9	4.1	.04
Model 3	Z	A	D,S	1,124.1	−558	1.9	0.1	.7
Model 4	D	A,Z	S	1,144.6	−568.3	18.6	20.6	<.0001
Model 5	S	A,Z	D	1,129.5	−560.8	3.5	5.6	.01
**Redness reference**		**1**	**D,S**	**1,175.5**	−**583.8**			
Model 6	R	R	D,S	1,174	−583.9	1.5	0.4	.5

Different logistic mixed‐effects models were compared to a reference (in bold) to test if the effects of astaxanthin (A), zeaxanthin (Z), dam (D), sire (S), and redness of the eggs (R) explain a significant part of the variance in embryo survival within (a) sham‐treated controls, (b) embryos exposed to low (NB 1:1,000) or (c) high nutrient broth concentrations (NB 1:500). Significant effects are highlighted.

**Figure 3:**
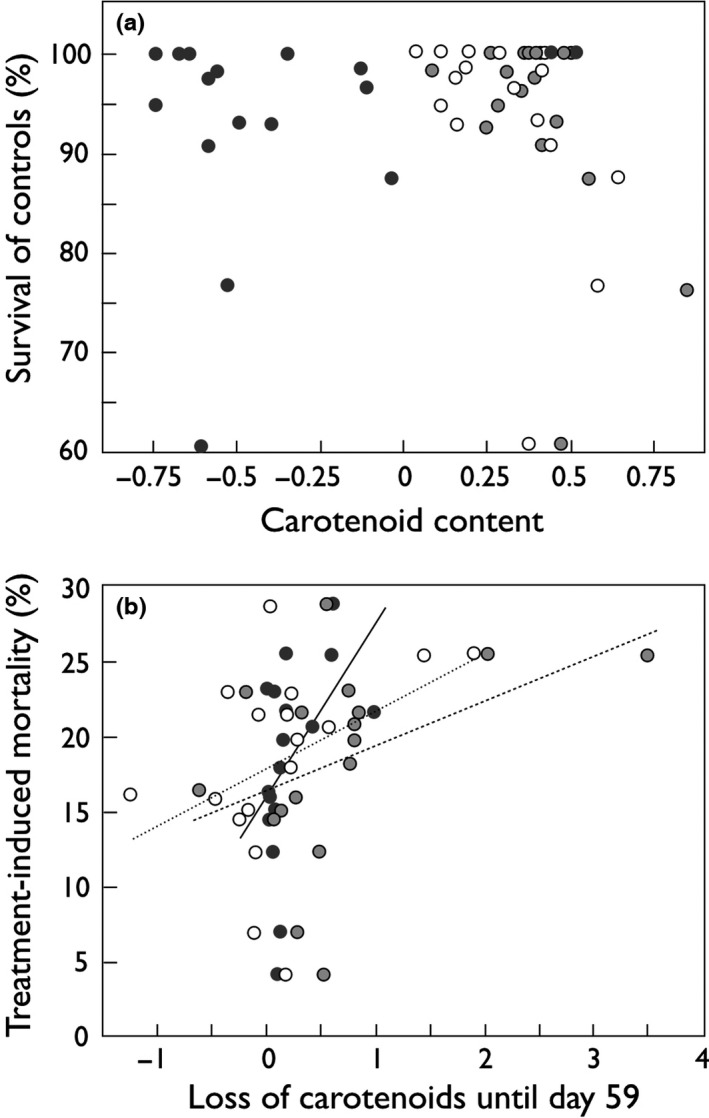
Relationship between embryo survival and egg carotenoid content. (a) Mean embryo survival until hatching per dam (means of maternal half‐sib groups) versus carotenoid contents of unfertilized eggs (log_10_‐transformed means per dam in μg/ml), and (b) treatment‐induced mortality; for example, mean survival per dam in sham‐treated controls minus mean survival in highest stress treatment (nutrient broth at 1:500) versus reduction of carotenoid content from day of fertilization until 14 days after treatment (in μg/ml). Astaxanthin: black symbols and nonhatched regression line; zeaxanthin: gray symbols and hatched regression line, lutein: open symbols and dotted regression line. See Tables 2 and 3 for statistics. Regression lines illustrate the direction of significant effects

**Figure 4:**
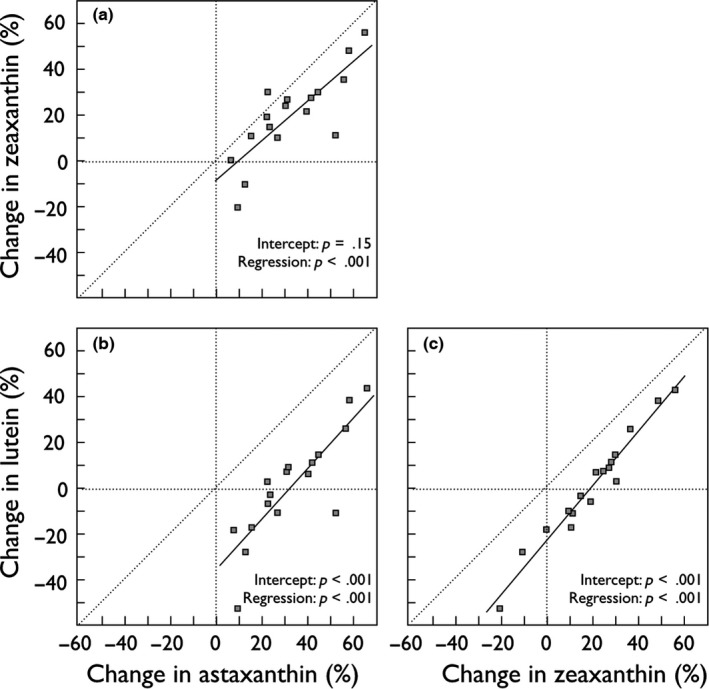
Changes of carotenoids during embryo development. The relative loss of astaxanthin, zeaxanthin, and lutein from fertilization to 14 days after incubation under the highest stress treatment (nutrient broth at 1:500). While the loss of astaxanthin and zeaxanthin was very similar (linear fit: *r* ^2^ = 0.67, intercept: *t *=* *−1.5, *p *=* *.15; regression: *t *=* *5.5, *p *<* *.0001), the loss of lutein correlated with the loss of the other two carotenoids but was less pronounced (linear fit to astaxanthin: *r* ^2^ = 0.72, intercept: *t *=* *−5.3, *p *<* *.0001; regression: *t *=* *6.2, *p *<* *.0001; linear fit to zeaxanthin: *r* ^2^ = 0.96, intercept: *t *=* *−13.2, *p *<* *.0001; regression: *t *=* *18.7, *p *<* *.0001). Nonhatched lines give the regressions, negative values indicate increased concentrations after 59 days of incubation

When carotenoid contents within the high nutrient broth treatment were replaced with the difference in carotenoid content before fertilization and 14 days after treatment, all individually analyzed carotenoids showed a significant effect on embryo survival (Table 3). The consumption of carotenoids was positively correlated to embryo mortality per maternal sib group (Figure 3). The losses of astaxanthin and zeaxanthin were qualitatively and quantitatively very similar among maternal sib groups (Figure 4a). The change of lutein content was also strongly correlated to the decrease of the other two carotenoids, but was significantly less pronounced (Figure 4b,c). Eight of 17 maternal sib groups even showed increased lutein levels after 59 days of incubation (the negative losses in Figure 4).

**Table 3:** Logistic mixed model regression testing the individual effects of carotenoid changes on embryo survival in the high nutrient broth treatment

Model	Effect tested	Model parameters		AIC	ln L	Likelihood ratio tests		
		Fixed	Random			δAIC	**χ** ^**2**^	*p*
a) Astaxanthin								
**Reference model**		**δA**	**D,S**	**1,128.8**	**−560.4**			
Model 1	δA	1	D,S	1,174	−583.9	45.2	47	<.0001
Model 2	D	δA	S	1,164.4	−579.2	35.6	37	<.0001
Model 3	S	δA	D	1,132.1	561.1	3.3	5.3	.02
b) Lutein								
**Reference model**		**δL**	**D,S**	**1,128.7**	**−560.33**			
Model 4	δL	1	D,S	1,174	−583.9	45.3	43.3	<.0001
Model 5	D	δL	S	1,163.9	−578.9	35.2	37.3	<.0001
Model 6	S	δL	D	1,132.1	−563.1	3.4	5.4	.02
c) Zeaxanthin								
**Reference model**		**δZ**	**D,S**	**1,127.4**	**−559.7**			
Model 1	δZ	1	D,S	1,174	−583.9	46.6	48.3	<.0001
Model 2	D	δZ	S	1,158	−576.1	30.6	32.6	<.0001
Model 3	S	δZ	D	1,130.9	−562.5	3.5	5.5	.02

Models were compared to a reference (in bold) analogous to Tables 1 and 2, a) astaxanthin, b) lutein, and c) zexanthin changes. The relationships of carotenoid changes and embryo survival are shown in Fig. 3b.

Despite the significant dam effects that we found, embryo survival under the different stress conditions could not be predicted by female origin (river Kiese or Müsche), size, weight, red coloration or darkness of the skin, and mean egg size (Table 4). Table 5 gives the estimated variance components for survival, and Table S5 gives the ones for hatching time.

**Table 4:** Logistic mixed model regressions testing the effects of dam characteristics on embryo survival

Model	Effect tested	Model parameters		AIC	ln L	Likelihood ratio tests		
		Fixed	Random			δAIC	**χ** ^**2**^	*p*
**Reference model**		**T**	**D,S**	**2,317.1**	**−1,153.5**			
Model 1	Origin	T	D,S,P	2,319	−1,153.5	1.9	0.04	.84
Model 2	Weight	T,W	D,S	2,318.5	−1,153.3	1.4	0.56	.45
Model 3	Length	T,L	D,S	2,318.4	−1,153.2	1.3	0.65	.42
Model 4	Red spots^a^	T,R1^a^	D,S	2,318.8	−1,153.4	1.7	0.33	.56
Model 5	Redness^b^	T,R2^b^	D,S	2,319	−1,153.5	1.9	0.1	.74
Model 6	Gray value	T,G	D,S	2,318	−1,153	0.9	1.1	.29
Model 7	Egg size	T,E	D,S	2,318.6	−1,153.3	1.5	0.5	.48
Interaction models								
Model 8	TxP	T	D,S	2,328.5	−1,153.3	11.4	0.54	.99
Model 9	TxW	T	D,S	2,322.2	−1,153.1	5.1	0.85	.84
Model 10	TxL	T	D,S	2,321.9	−1,152.9	4.8	1.2	.75
Model 11	TxR1^a^	T	D,S	2,321.6	−1,152.8	4.5	1.53	.67
Model 12	TxR2^b^	T	D,S	2,322.9	−1,153.5	5.8	0.13	.99
Model 13	TxG	T	D,S	2,319	−1,151.5	1.9	4.01	.25
Model 14	TxE	T	D,S	2,320.8	−1,152.4	3.7	2.3	.5

Models were compared to a reference (in bold) analogous to Tables 1 and 2 (i.e., T = treatment, D = dam, S = sire). Female origin (P = population) was treated as a random effect while all other characteristics (W = weight, L = length, R1 = proportional area of red spots on skin, R2 = relative redness of skin, G = darkness of the skin, and E = egg size) were treated as fixed effects. Redness was measured either as ^a^proportional area of red spots or as ^b^relative redness of skin. See text for its calculation.

**Table 5:** Maternal variance components for embryo survival

Environment	*V*_DAM_	*V*_A_	*V*_MENV_	*V*_ASTA_	*V*_CONS_
a) Control	2.57 (0.05)***	2.56 (0.21)**	1.94 (0.07)***	1.35 (0.04)	–
b) NB 1:1,000	1.14 (0.03)***	0.72 (0.11)*	0.97 (0.04) ***	0.05 (0.01)	–
c) NB 1:500	0.43 (0.01)***	0.36 (0.06)*	0.34 (0.02)***	0.003 (0.007)*	–
d) NB 1:500	0.38 (0.01)***	0.36 (0.06)**	0.29 (0.02)***	‐	0.04 (0.01)***

*V*_DAM_, total maternal variance; *V*_A_, additive genetic variance; *V*_MENV_, maternal environmental variance; *V*_ASTA_, variance explained by astaxanthin content in unfertilized eggs; *V*_CONS_, variance explained by astaxanthin consumption. Numbers in parentheses indicate standard deviations. Asterisks show significance values in Tables 2 and 3: *<.05, *^*^<.01, *^**^<.001.

Time until hatching was on average (±*SD*; based on means per maternal sib groups; i.e., *N* = 18): control = 68.46 ± 0.8 days (d), NB 1:1,000 = 68.61 ± 0.7 days, NB 1:500 = 68.13 ± 0.7 days. Embryos hatched significantly later at the low nutrient broth concentration and significantly earlier at the high nutrient broth concentration relative to the control (Tables S6 and S7; Figure S1a). Hatching times also differed between the offspring of different dams and sires, both, in the overall model (Tables S6 and S7) and within treatment groups (Tables S8 and S9). Within the high nutrient broth treatment, there was no evidence for a significant relationship between carotenoid changes and hatching times (Figure S1b; *r* always <.14, *N* = 17, *p* always >.5). The results of carotenoid contents for astaxanthin and zeaxanthin are shown in Tables S6 and S8, and astaxanthin and lutein are shown in Tables S7 and S9. Embryos from bigger eggs hatched earlier in the low nutrient broth treatment group; that is, this is reflected in the significant interaction term of model 14 (Table S10).

## 4 DISCUSSION

### 4.1 Natural carotenoid content in brown trout eggs

Of the eight pigment molecules that were targeted, only astaxanthin, lutein, and zeaxanthin could be found above a critical detection threshold in eggs of all wild brown trout that were sampled from two different natural rivers. These three xanthophylls are potent antioxidant compounds produced by plants (Goodwin, 1984). Astaxantin and lutein are commonly found in skins and eggs of fish (e.g., Kodric‐Brown, 1989; Tyndale et al., 2008; Wedekind, Meyer, Frischknecht, Niggli, & Pfander, 1998), while zeaxanthin, a stereoisomer of lutein (Khachik, Askin, & Lai, 1998), seems rarely targeted in analytical studies. Hence, we are reporting the results including zeaxanthin and show the analogous results on lutein in the supplement. Fish acquire these carotenoids through their natural diet; that is, mostly from accumulated carotenoids in crustaceans and algae (Choubert et al., 1995). Then, they are reallocated to their eggs and other tissues. All three carotenoids turned out to be correlated to egg redness, as expected positively in the case of astaxanthin and zeaxanthin (Berman et al., 2015), and negatively in the case of lutein (that colors tissues yellow; Wedekind et al., 1998). Moreover, astaxanthin contents were neither correlated to zeaxanthin nor to lutein contents in unfertilized eggs, while the contents of the two stereoisomers lutein and zeaxanthin were highly correlated. Due to the collinearity between lutein and zeaxanthin, we could not include them in the same model investigating their effects on embryo viability.

### 4.2 The effects of environmental pollution

Our stress treatment turned out to be effective: It proved detrimental to the fish and caused embryo mortality as in Jacob et al. (2010) who studied embryo performance of another life‐history form of brown trout from Lake Geneva (*Salmo trutta* morpha *lacustris*). It also caused a divergent effect on embryo hatching times among the different treatment groups: At low nutrient broth concentrations, the embryos hatched comparatively late, potentially as a stress response, while at the high nutrient broth concentration the embryos hatched even before the control group. Early hatching can be interpreted as way for the embryos to escape the environmental threat (Wedekind & Müller, 2005), while delayed hatching is generally considered disadvantageous for the larvae (Clark, Pompini, da Cunha, & Wedekind, 2014).

Adding nutrient broth simulates organic pollution (Wedekind et al., 2010). Hence, this is a rather unspecific stress as compared to, for example, the addition of a specific pathogen. Organic pollution has been shown to affect the virulence of egg‐associated microbial communities (Wedekind et al., 2010) and their composition and functional pathways (Wilkins et al., 2016). It induces embryo mortality that can be linked to the allelic diversity in the major histocompatibility complex (Jacob et al., 2010). Therefore, we expected additive genetic variance in the stress response, which was confirmed by the significant sire effects we found here.

### 4.3 Parental effects

Additive genetic variance for stress tolerance is typically found in experiments on singly raised embryos that have been produced by full‐factorial breeding. Such genetic effects can be interpreted as the overall genetic quality of the embryo; for example, its immune competence or its genetic load (Neff & Pitcher, 2005). The fitness‐associated traits include, for example, tolerance to uncontrolled epidemics or nonspecified stressors (e.g., Evans, Neff, & Heath, 2010; Pitcher & Neff, 2007; Wedekind, Müller, & Spicher, 2001), specific bacterial infections (Clark et al., 2014; von Siebenthal, Jacob, & Wedekind, 2009), chemical pollution (Brazzola, Chèvre, & Wedekind, 2014), or pollution by nanoparticles (Clark, Pompini, Uppal, & Wedekind, 2016). The present study adds organic pollution to this list.

Experiments based on full‐factorial breeding typically also find significant dam effects on stress tolerance (e.g., Aykanat et al., 2012; Evans et al., 2010; Pitcher & Neff, 2007; Wedekind et al., 2001). Dam effects are a mixture of additive genetic effects, maternal environmental effects, and their interactions (Lynch & Walsh, 1998). Our full‐factorial breeding design and the large number of dams and sires we used allowed us not only to estimate paternal additive genetic effects, but also maternal environmental effects on survival. The latter are calculated by subtracting the significant sire effects from the total dam effects (Lynch & Walsh, 1998), based on the assumption of no interaction between maternal genetic and maternal environmental effects.

### 4.4 Effects of carotenoids

The total maternal environmental effects we found here were up to 5.4 times larger than additive genetic effects. Despite these large maternal effects, female origin (i.e., the two streams), female skin coloration, variance in female size and weight, and variance in mean egg size among females did not explain the variation in embryo stress tolerance. Moreover, we found no significant effects of carotenoid content in unfertilized eggs on the tolerance of embryos to the experimentally induced stress; that is, on embryo survival or hatching time. We cannot exclude the possibility that such correlations exist and would be detectable in a study based on more statistical power. However, a significant part of the dam effects was explained by the consumption of all three carotenoids. The change in carotenoids correlated positively with mortality per sib group. Sib groups that suffered more from the induced stress consumed on average more carotenoids. Eight of the 17 maternal sib groups showed increased lutein contents at day 59 of embryogenesis as compared to day zero. It seems unlikely that this observation is due to measurement error, given the good correlations between the changes of all three carotenoid contents. Instead, the observed change in lutein could be due to isomerization, possibly from zeaxanthin (Khachik et al., 1998; the corresponding *r* ^2^ between the changes in lutein and zeaxanthin was very close to 1.0) but probably also from astaxanthin, given the strong correlations in the relative changes of all three carotenoids.

Carotenoids either prevent stress (“passive immunity”; Anbazahan et al., 2014) or are used up during the physiological defense against stress (Palace & Werner, 2006). If they protect against stress; that is, if carotenoids would be important at the first line of defense, we would expect a positive correlation between carotenoid content and stress tolerance, and no correlation between stress tolerance and carotenoid consumption (Brown et al., 2016). If they are only used up in defense of stress, the primary susceptibility of a sib group would be given by noncarotenoid‐based factors; for example, genetic factors. Sib groups that vary at these noncarotenoid‐based factors would then also show variation in their stress response. In this situation, the role of carotenoids would be to mitigate the stress. The following types of correlations are possible: (1) A negative correlation between carotenoid content in unfertilized eggs and later embryo mortality combined with a positive correlation between embryo mortality and carotenoid consumption would suggest that carotenoid consumption helps fight the stress, but that the defense is not 100% effective and that carotenoids content is limiting. (2) No correlation between carotenoid content in unfertilized eggs and later embryo mortality combined with a positive correlation between mortality and carotenoid consumption would indicate that carotenoid consumption helps fight the stress, but that the defense is not 100% effective and carotenoid contents were not limiting under the given conditions. If the defense was fully effective, we would only see variation in carotenoid consumption but not in mortality. What we observed was that carotenoid content seemed not be limiting yet (i.e., we found no significant correlation between carotenoid content of unfertilized eggs and embryo mortality), while carotenoid consumption was positively correlated with mortality per maternal sib group. Due to the collinearity of carotenoid consumption, we cannot point out which carotenoid was particularly affected or whether the combination of different carotenoid compounds matter. We conclude that sib groups vary in their susceptibility to the type of stress we inflicted (the organic pollution) mainly because of noncarotenoid‐based factors (e.g., egg membrane characteristics or embryo genetics) and that carotenoids are then used up in defense against the stress.

## DATA ACCESSIBILITY

Data on embryo performance, parental characteristics, and carotenoid measurements, as well as R‐scripts used for the analysis are deposited on the Dryad repository https://doi.org/10.5061/dryad.0mr42.

## CONFLICT OF INTEREST

There are no conflicts of interest.

## AUTHOR CONTRIBUTIONS

LW and CW designed the project. LW, LMC, and CW sampled the fish, did the in vitro fertilizations, distributed the eggs to plates, and discussed the data analysis. LMC took the biometrical and color measures. All manipulations and measurements on the embryonated eggs were performed by LW. GG, AV, and LW did the chemical analyses. LW and CW wrote a first version of the manuscript that was then critically revised by all authors.
