# Supplementary material for: Environmental stress linked to consumption of maternally derived carotenoids in brown trout embryos (Salmo trutta)
Source: Ecol Evol. 2017 Jun 2;7(14):5082–93. doi: 10.1002/ece3.3076 (PMC5528241; doi:10.1002/ece3.3076)

**Supplementary Material**

**Environmental stress linked to consumption of maternally derived carotenoids in brown trout embryos**

**(*Salmo trutta*)**

Laetitia G.E. Wilkins^a,b*^, Lucas Marques da Cunha^a^, Gaëtan Glauser^c^, Armelle Vallat^c^, and Claus Wedekind^a^

^a^Department of Ecology and Evolution, Biophore, University of Lausanne, 1015 Lausanne, Switzerland

^b^Department of Environmental Sciences, Policy & Management, 130 Mulford Hall #3114, University of California, Berkeley, CA 94720, USA

^c^Neuchâtel Platform of Analytical Chemistry, University of Neuchâtel, Avenue de Bellevaux 51, 2000 Neuchâtel, Switzerland

^*^Correspondence to L. Wilkins, Tel: +41 21 692 42 19; Fax: +41 21 692 42 65; email: [laetitia.wilkins@unil.ch](mailto:laetitia.wilkins@unil.ch)

Table S1: Adult brown trout used for the two breeding blocks and the carotenoid contents in their eggs.

|  |  |  | **Carotenoids^b^ in unfertilized eggs** | | |  | **Carotenoids after high-stress treatment** | | |  | |  |
| --- | --- | --- | --- | --- | --- | --- | --- | --- | --- | --- | --- | --- |
| **ID** | **Block**^a^ | **River** | **Astaxanthin** | **Zeaxanthin** | **Lutein** |  | **Astaxanthin** | **Zeaxanthin** | **Lutein** | **Comments** |  |  |
| Dam 1 | 1 | Kiese | 1520.8 | 6406.4 | 7701.7 |  | 531.5 | 2826.1 | 4350.7 | bacterial communities**^c^** | | |
| Dam 2 | 1 | Kiese | 509.1 | 12695.5 | 6641.4 |  | 213.8 | 6558.1 | 4106.6 | bacterial communities**^c^** | | |
| Dam 3 | 1 | Kiese | 1308.2 | 3925.8 | 3812.4 |  | 572.0 | 2515.1 | 2817.5 | bacterial communities**^c^** | | |
| Dam 4 | 1 | Kiese | 418.9 | 5351.6 | 4240.9 |  | 381.3 | 6449.9 | 6469.9 | bacterial communities**^c^** | | |
| Dam 5 | 1 | Kiese | 302.9 | 3437.6 | 6174.5 |  | 265.2 | 3799.6 | 2885.8 | bacterial communities**^c^** | | |
| Dam 6 | 1 | Kiese | 5413.1 | 5468.8 | 3361.9 |  | 3749.6 | 4016.0 | 3071.4 | bacterial communities**^c^** | | |
| Dam 7 | 1 | Kiese | 4742.9 | 3300.8 | 1900.7 |  | 3697.6 | 2310.9 | 1845.8 | bacterial communities**^c^** | | |
| Dam 8 | 1 | Kiese | 547.8 | 3222.7 | 2565.4 |  | 508.4 | 3218.2 | 3032.3 | bacterial communities**^c^** | | |
| Dam 9 | 1 | Kiese | 1263.1 | 3652.4 | 4586.9 |  | 970.6 | 3110.0 | 4731.6 | - | | |
| Dam 10 | 1 | Kiese | 464.0 | 2128.9 | 2658.8 |  | 340.9 | 1920.1 | 2944.4 | - | | |
| Dam 11 | 1 | Kiese | - | - | - |  | - | - | - | samples accidentially lost | | |
| Dam 12 | 1 | Kiese | 747.5 | 5586.0 | 4669.3 |  | 520.0 | 4218.8 | 4321.4 | - | | |
| Sires 1-7 | 1 | Kiese | - | - | - |  | - | - | - | bacterial communities**^c^** | | |
| Sire 8 | 1 | Kiese | - | - | - |  | - | - | - | - | | |
| Dam 13 | 2 | Müsche | 360.9 | 4336.0 | 2318.2 |  | 173.3 | 3826.7 | 2568.4 | - | | |
| Dam 14 | 2 | Müsche | 391.2 | 4121.2 | 4537.5 |  | 236.3 | 3231.7 | 4243.3 | - | | |
| Dam 15 | 2 | Müsche | 438.2 | 4414.1 | 2505.0 |  | 340.9 | 3569.8 | 2671.0 | - | | |
| Dam 16 | 2 | Müsche | 304.2 | 4492.3 | 2818.1 |  | 168.7 | 3150.6 | 2412.2 | - | | |
| Dam 17 | 2 | Müsche | 683.1 | 5156.3 | 4510.0 |  | 399.2 | 3718.5 | 4013.8 | - | | |
| Dam 18 | 2 | Müsche | 444.0 | 4589.9 | 4872.6 |  | 377.3 | 4097.1 | 5703.3 | - | | |
| Sires 9-13 | 2 | Müsche | - | - | - |  | - | - | - | - | | |

^a^ Full-factorial breeding blocks: F1-F12 x M1-M8 (= 96 half-sib groups; river Kiese) and F13-F18 x M9-M15 (= 30 half-sib groups; river Müsche). ^b^ Carotenoid contents measured in nM in five unfertilized eggs. ^c^ Bacterial communities were analysed on embryos of these parents and compared to embryo survival in the respective 56 half-sib groups (see Wilkins, Fumagalli & Wedekind, in press).

Table S2: Analysis of variance (ANOVA) table for egg redness and egg sizes among females.

| **Model terms** | **d.f.** | **SS** | **F** | **R^2^** | ***p*** |
| --- | --- | --- | --- | --- | --- |
| *a) Egg redness* |  |  |  |  |  |
| Female | 17 | 6529 | 25.8 | 0.87 | < 0.0001 |
| Residuals | 72 | 598 |  |  |  |
| Total | 90 | 7127 |  |  |  |
| *b) Egg sizes* |  |  |  |  |  |
| Female | 17 | 14594 | 405 | 0.9 | < 0.0001 |
| Residuals | 72 | 152 |  |  |  |
| Total | 90 | 14746 |  |  |  |

Table S3: Alternative likelihood ratio tests of logistic mixed model regressions on trout embryo survival with zeaxanthin contents replaced by lutein contents.

Different logistic mixed effects models were compared to a reference (in bold) to test if the effects of treatment (T), astaxanthin (A), lutein (L), dam (D), sire (S), population (P), redness of the eggs (R), and the interactions dam x sire (DxS), treatment x dam (TxD), treatment x sire (TxS), treatment x astaxanthin (TxA), and treatment x lutein (TxL) explain a significant part of the variance in embryo survival (carotenoid contents were measured in five unfertilized eggs). Significant effects are highlighted.

|  |  | Model parameters | |  |  | Likelihood ratio tests | | |
| --- | --- | --- | --- | --- | --- | --- | --- | --- |
| Model | Effect tested | Fixed | Random | AIC | ln L | δAIC | **χ^2^** | *p* |
| **Reference model** | | **T,A,L** | **D,S** | **2221.1** | **-1103.5** |  |  |  |
| Model 1 | T | A,L | D,S | 2377.2 | -1183.6 | 156.1 | 160 | <0.0001 |
| Model 2 | A | T,L | D,S | 2219.1 | -1103.6 | 2 | 0.03 | 0.87 |
| Model 3 | L | T,A | D,S | 2223.1 | -1105.1 | 2 | 2.9 | 0.06 |
| Model 4 | D | T,A,L | S | 2353 | -1170.5 | 131.9 | 133.9 | <0.0001 |
| Model 5 | S | T,A,L | D | 2244.8 | -1116.4 | 23.7 | 25.7 | <0.001 |
| Model 6 | P | T,A,L | D,S,P | 2223 | -1103.5 | 1.9 | 0.11 | 0.7 |
|  |  |  |  |  |  |  |  |  |
| **Redness reference** | | **T** | **D,S** | **2317.1** | **-1153.5** |  |  |  |
| Model 7 | R | T, R | D,S | 2319.1 | -1153.5 | 2 | 0.02 | 0.86 |
|  |  |  |  |  |  |  |  |  |
| Interaction models: | |  |  |  |  |  |  |  |
| Model 8 | DxS | T,A,L | D,S | 2383.4 | -1092.7 | 160.7 | 21.3 | 1 |
| Model 9 | TxD | T,A,L | S | 2209.8 | -1092.9 | 11.3 | 21.3 | 0.0007 |
| Model 10 | TxS | T,A,L | D | 2228.5 | -1102.2 | 7.4 | 2.6 | 0.8 |
| Model 11 | TxA | T,L | D,S | 2213 | -1097.5 | 8.1 | 12 | 0.002 |
| Model 12 | TxL | T,A | D,S | 2219.9 | -1100 | 1.2 | 3.7 | 0.06 |

Table S4: Alternative likelihood ratio tests of logistic mixed model regressions on trout embryo survival within treatments with zeaxanthin contents replaced by lutein contents.

Different logistic mixed effects models were compared to a reference (in bold) to test if the effects of astaxanthin (A), lutein (L), dam (D), sire (S), and redness of the eggs (R) explain a significant part of the variance in embryo survival within (a) sham-treated controls, (b) embryos exposed to low (NB 1:1000) or (c) high nutrient broth concentrations (NB 1:500).

|  |  | Model parameters | |  |  | Likelihood ratio tests | | |
| --- | --- | --- | --- | --- | --- | --- | --- | --- |
| Model | Effect tested | Fixed | Random | AIC | ln L | δAIC | **χ^2^** | *p* |
| a)    Controls | |  |  |  |  |  |  |  |
| **Reference model** | | **A,L** | **D,S** | **1127.1** | **-558.8** |  |  |  |
| Model 1 | A | L | D,S | 1125.3 | -557.7 | 1.8 | 0.3 | 0.6 |
| Model 3 | L | A | D,S | 1128.9 | -560.4 | 1.8 | 3.8 | 0.06 |
| Model 4 | D | A,L | S | 1150 | -571 | 22.9 | 24.9 | <0.0001 |
| Model 5 | S | A,L | D | 1130.5 | 561.23 | 3.4 | 5.4 | 0.02 |
| **Redness reference** | | **1** | **D,S** | **1175.5** | **-583.8** |  |  |  |
| Model 6 | R | R | D,S | 1174 | -583.9 | 1.5 | 0.4 | 0.5 |
|  |  |  |  |  |  |  |  |  |
| b)   NB 1:1000 | |  |  |  |  |  |  |  |
| **Reference model** | | **A,L** | **D,S** | **740.5** | **-365.3** |  |  |  |
| Model 1 | A | L | D,S | 738.9 | -365.5 | 1.6 | 0.4 | 0.54 |
| Model 3 | L | A | D,S | 741.9 | -365.9 | 1.4 | 3.4 | 0.07 |
| Model 4 | D | A,L | S | 785.5 | -388.7 | 45 | 46.9 | <0.0001 |
| Model 5 | S | A,L | D | 744.9 | -368.5 | 4.4 | 6.4 | 0.01 |
| **Redness reference** | | **1** | **D,S** | **752.2** | **-373.1** |  |  |  |
| Model 6 | R | R | D,S | 753.8 | -372.9 | 1.6 | 0.3 | 0.7 |
|  |  |  |  |  |  |  |  |  |
| c)    NB 1:500 | |  |  |  |  |  |  |  |
| **Reference model** | | **A,L** | **D,S** | **384.1** | **-187** |  |  |  |
| Model 1 | A | L | D,S | 386.3 | -189.2 | 2.2 | 4.2 | 0.04 |
| Model 3 | L | A | D,S | 385 | -187.2 | 0.9 | 1.6 | 0.07 |
| Model 4 | D | A,L | S | 412.6 | -202.3 | 28.5 | 30.5 | <0.0001 |
| Model 5 | S | A,L | D | 392.2 | -192.1 | 8.1 | 10.1 | 0.001 |
| **Redness reference** | | **1** | **D,S** | **422.6** | **-207.3** |  |  |  |
| Model 6 | R | R | D,S | 420.7 | -207.3 | 1.9 | 0.09 | 0.76 |

Table S5: Maternal variance components for hatching times.

V_DAM_ = total maternal variance, V_A_ = additive genetic variance, V_MENV_ = maternal environmental variance, V_ASTA_ = variance explained by astaxanthin content in unfertilized eggs, V_CONS_ = variance explained by astaxanthin consumption. Numbers in parentheses indicate standard deviations. Asterisks show significance values in Tables S6 & S8: * < 0.05, ** < 0.01, *** < 0.001.

| **Environment** | **V_DAM_** | **V_A_** | **V_MENV_** | **V_ASTA_** | **V_CONS_** |
| --- | --- | --- | --- | --- | --- |
| a) Control | 2.26(0.04)^***^ | 2.06(0.03)^***^ | 1.75(0.01) ^***^ | 0.002(0.002) | - |
| b) NB 1:1000 | 1.68(0.006)^***^ | 1.36(0.08)^***^ | 1.34(0.01)^***^ | 0.02(0.004) | - |
| c) NB 1:500 | 2.88(0.01)^***^ | 2.92(0.03)^***^ | 2.15(0.01) ^***^ | 0.003(0.002) | - |
| d) NB 1:500 | 2.9(0.02)^***^ | 2.91(0.03)^***^ | 2.18(0.03) ^***^ | - | 0.006(0.003) |

Table S6: Likelihood ratio tests of linear mixed model regressions on time until hatching.

Different linear mixed effects models were compared to a reference (in bold) to test if the effects of treatment (T), astaxanthin (A), zeaxanthin (Z), dam (D), sire (S), population (P), and redness of the eggs (R) explain a significant part of the variance in time from fertilization until hatching (carotenoid contents were measured in five unfertilized eggs). Significant effects are highlighted.

|  |  | Model parameters | |  |  | Likelihood ratio tests | | |
| --- | --- | --- | --- | --- | --- | --- | --- | --- |
| Model | Effect tested | Fixed | Random | AIC | ln L | δAIC | **χ^2^** | *p* |
| **Reference model** | | **T,A,Z** | **D,S** | **11139** | **-5561.5** |  |  |  |
| Model 1 | T | A,Z | D,S | 11179 | -5583.6 | 40 | 44.2 | <0.0001 |
| Model 2 | A | T,Z | D,S | 11137 | -5561.5 | 2 | 0.001 | 0.98 |
| Model 3 | Z | T,A | D,S | 11137 | -5561.5 | 2 | 0.001 | 0.98 |
| Model 4 | D | T,A,Z | S | 11558 | -5771.9 | 419 | 420.8 | <0.0001 |
| Model 5 | S | T,A,Z | D | 11549 | -5767.6 | 410 | 412.3 | <0.0001 |
| Model 6 | P | T,A,Z | D,S,P | 11141 | -5561.5 | 2 | 0 | 1 |
|  |  |  |  |  |  |  |  |  |
| **Redness reference** | | **T** | **D,S** | **11672** | **-5828.8** |  |  |  |
| Model 7 | R | T, R | D,S | 11714 | -5828.8 | 42 | 0.04 | 0.84 |
|  |  |  |  |  |  |  |  |  |
| Interaction models: | |  |  |  |  |  |  |  |
| Model 8 | DxS | T,A,Z | D,S | 11131 | -5560 | 8 | 21.3 | 1 |
| Model 9 | TxD | T,A,Z | S | 11026 | -5500.1 | 113 | 122.7 | <0.0001 |
| Model 10 | TxS | T,A,Z | D | 11023 | -5498.6 | 116 | 125.7 | <0.0001 |
| Model 11 | TxA | T,Z | D,S | 11116 | -5547.8 | 23 | 13.3 | 0.03 |
| Model 12 | TxZ | T,A | D,S | 11134 | -5556.9 | 5 | 4.1 | 0.2 |

Table S7: Alternative likelihood ratio tests of linear mixed model regressions on time until hatching with zeaxanthin contents replaced by lutein contents.

Different linear mixed effects models were compared to a reference (in bold) to test if the effects of treatment (T), astaxanthin (A), lutein (L), dam (D), sire (S), population (P), and redness of the eggs (R) explain a significant part of the variance in time from fertilization until hatching (carotenoid contents were measured in five unfertilized eggs). Significant effects are highlighted.

|  |  | Model parameters | |  |  | Likelihood ratio tests | | |
| --- | --- | --- | --- | --- | --- | --- | --- | --- |
| Model | Effect tested | Fixed | Random | AIC | ln L | δAIC | **χ^2^** | *p* |
| **Reference model** | | **T,A,L** | **D,S** | **11139** | **-5561.5** |  |  |  |
| Model 1 | T | A,L | D,S | 11179 | -5583.6 | 40 | 44.2 | <0.0001 |
| Model 2 | A | T,L | D,S | 11137 | -5561.5 | 2 | 0.003 | 0.95 |
| Model 3 | L | T,A | D,S | 11137 | -5561.5 | 2 | 0.003 | 0.95 |
| Model 4 | D | T,A,L | S | 11553 | -5769.6 | 414 | 416.2 | <0.0001 |
| Model 5 | S | T,A,L | D | 11549 | -5767.6 | 410 | 412.3 | <0.0001 |
| Model 6 | P | T,A,L | D,S,P | 11141 | -5561.5 | 2 | 0 | 1 |
|  |  |  |  |  |  |  |  |  |
| **Redness reference** | | **T** | **D,S** | **11672** | **-5828.8** |  |  |  |
| Model 7 | R | T, R | D,S | 11670 | -5828.8 | 2 | 0.03 | 0.84 |
|  |  |  |  |  |  |  |  |  |
| Interaction models: | |  |  |  |  |  |  |  |
| Model 8 | DxS | T,A,L | D,S | 11134 | -5560.3 | 5 | 19.3 | 1 |
| Model 9 | TxD | T,A,L | S | 11026 | -5500 | 113 | 123 | <0.0001 |
| Model 10 | TxS | T,A,L | D | 11023 | -5498.6 | 116 | 125.8 | <0.0001 |
| Model 11 | TxA | T,L | D,S | 11116 | -5547.8 | 23 | 27 | 0.02 |
| Model 12 | TxL | T,A | D,S | 11133 | -5556.7 | 6 | 2.7 | 0.17 |

Table S8: Likelihood ratio tests of linear mixed model regressions on time until hatching within treatment groups.

Different linear mixed effects models were compared to a reference (in bold) to test if the effects of astaxanthin (A), zeaxanthin (Z), dam (D), sire (S), and redness of the eggs (R) explain a significant part of the variance in hatching time within (a) sham-treated controls, (b) embryos exposed to low (NB 1:1000) or (c) high nutrient broth concentrations (NB 1:500).

|  |  | Model parameters | |  |  | Likelihood ratio tests | | |
| --- | --- | --- | --- | --- | --- | --- | --- | --- |
| Model | Effect tested | Fixed | Random | AIC | ln L | δAIC | **χ^2^** | *p* |
| a)    Controls | |  |  |  |  |  |  |  |
| **Reference model** | | **A,Z** | **D,S** | **3484** | **-1736** |  |  |  |
| Model 1 | A | Z | D,S | 3482 | -1736 | 2 | 0.01 | 0.9 |
| Model 3 | Z | A | D,S | 3482 | -1736 | 2 | 0.01 | 0.9 |
| Model 4 | D | A,Z | S | 3662 | -1826 | 178 | 180 | <0.0001 |
| Model 5 | S | A,Z | D | 3671 | -1830 | 187 | 189 | <0.0001 |
| **Redness reference** | | **1** | **D,S** | **3673.4** | **-1831.7** |  |  |  |
| Model 6 | R | R | D,S | 3671.4 | -1831.7 | 2 | 0.05 | 0.82 |
|  |  |  |  |  |  |  |  |  |
| b)   NB 1:1000 | |  |  |  |  |  |  |  |
| **Reference model** | | **A,Z** | **D,S** | **3584.8** | **-1786.4** |  |  |  |
| Model 1 | A | Z | D,S | 3583.5 | -1786.8 | 1.3 | 0.74 | 0.38 |
| Model 3 | Z | A | D,S | 3583.2 | -1786.6 | 1.6 | 0.42 | 0.51 |
| Model 4 | D | A,Z | S | 3707.9 | -1848.9 | 123.1 | 125.1 | <0.0001 |
| Model 5 | S | A,Z | D | 3687.1 | -1838.6 | 102.3 | 104.4 | <0.0001 |
| **Redness reference** | | **1** | **D,S** | **3756** | **-1873** |  |  |  |
| Model 6 | R | R | D,S | 3754.2 | -1873.1 | 1.8 | 0.3 | 0.6 |
|  |  |  |  |  |  |  |  |  |
| c)    NB 1:500 | |  |  |  |  |  |  |  |
| **Reference model** | | **A,Z** | **D,S** | **3913** | **-1950.5** |  |  |  |
| Model 1 | A | Z | D,S | 3911.3 | -1950.6 | 1.7 | 0.23 | 0.62 |
| Model 3 | Z | A | D,S | 3911.1 | -1950.5 | 1.9 | 0.03 | 0.86 |
| Model 4 | D | A,Z | S | 4054.2 | -2022.1 | 141.2 | 143.1 | <0.0001 |
| Model 5 | S | A,Z | D | 4068.5 | -2029.2 | 155.5 | 157.5 | <0.0001 |
| **Redness reference** | | **1** | **D,S** | **4090.6** | **-2040.3** |  |  |  |
| Model 6 | R | R | D,S | 4088.6 | -2040.3 | 2 | 0.02 | 0.9 |

Table S9: Alternative likelihood ratio tests of linear mixed model regressions on time until hatching within treatment groups with zeaxanthin contents replaced by lutein contents.

Different linear mixed effects models were compared to a reference (in bold) to test if the effects of astaxanthin (A), lutein (L), dam (D), sire (S), and redness of the eggs (R) explain a significant part of the variance in hatching time within (a) sham-treated controls, (b) embryos exposed to low (NB 1:1000) or (c) high nutrient broth concentrations (NB 1:500).

|  |  | Model parameters | |  |  | Likelihood ratio tests | | |
| --- | --- | --- | --- | --- | --- | --- | --- | --- |
| Model | Effect tested | Fixed | Random | AIC | ln L | δAIC | **χ^2^** | *p* |
| a)    Controls | |  |  |  |  |  |  |  |
| **Reference model** | | **A,L** | **D,S** | **3484.1** | **-1736** |  |  |  |
| Model 1 | A | L | D,S | 3482.1 | -1736 | 2 | 0.02 | 0.89 |
| Model 3 | L | A | D,S | 3482.8 | -1736.4 | 1.3 | 0.74 | 0.38 |
| Model 4 | D | A,L | S | 3666 | -1828.4 | 181.9 | 184.8 | <0.0001 |
| Model 5 | S | A,L | D | 3671.2 | -1830.6 | 187.1 | 189.1 | <0.0001 |
| **Redness reference** | | **1** | **D,S** | **3673.4** | **-1831.7** |  |  |  |
| Model 6 | R | R | D,S | 3671.4 | -1831.7 | 2 | 0.05 | 0.82 |
|  |  |  |  |  |  |  |  |  |
| b)   NB 1:1000 | |  |  |  |  |  |  |  |
| **Reference model** | | **A,L** | **D,S** | **3584.9** | **-1786.4** |  |  |  |
| Model 1 | A | L | D,S | 3583.5 | -1786.8 | 1.4 | 0.64 | 0.42 |
| Model 3 | L | A | D,S | 3583.2 | -1786.6 | 1.7 | 0.33 | 0.56 |
| Model 4 | D | A,L | S | 3710.8 | -1850.4 | 125.9 | 127.8 | <0.0001 |
| Model 5 | S | A,L | D | 3687.2 | -1838.6 | 102.3 | 104.36 | <0.0001 |
| **Redness reference** | | **1** | **D,S** | **3756** | **-1873** |  |  |  |
| Model 6 | R | R | D,S | 3754.2 | -1873.1 | 1.8 | 0.3 | 0.6 |
|  |  |  |  |  |  |  |  |  |
| c)    NB 1:500 | |  |  |  |  |  |  |  |
| **Reference model** | | **A,L** | **D,S** | **3913** | **-1950.5** |  |  |  |
| Model 1 | A | L | D,S | 3911.3 | -1950.6 | 1.7 | 0.22 | 0.63 |
| Model 3 | L | A | D,S | 3911.1 | -1950.5 | 1.9 | 0.03 | 0.86 |
| Model 4 | D | A,L | S | 4054.1 | -2022 | 141.1 | 143.4 | <0.0001 |
| Model 5 | S | A,L | D | 4068.6 | -2029.3 | 155.6 | 157.5 | <0.0001 |
| **Redness reference** | | **1** | **D,S** | **4090.6** | **-2040.3** |  |  |  |
| Model 6 | R | R | D,S | 4088.6 | -2040.3 | 2 | 0.01 | 0.9 |

Table S10: Logistic mixed model regressions testing the effects of dam characteristics on hatching time.

Models were compared to a reference (in bold) analogous to Tables 1, 2 & 4 (i.e., T = treatment, D = dam, S = sire). Female origin (P = population) was treated as a random effect while all other characteristics (W = weight, L = length, R1 = proportional area of red spots on skin, R2 = relative redness of skin, G = darkness of the skin, and E = egg size) were treated as fixed effects. Significant effects are highlighted.

|  |  | Model parameters | |  |  | Likelihood ratio tests | | |
| --- | --- | --- | --- | --- | --- | --- | --- | --- |
| Model | Effect tested | Fixed | Random | AIC | ln L | δAIC | **χ^2^** | *p* |
| **Reference model** | | **T** | **D,S** | **11670** | **-5828.8** |  |  |  |
| Model 1 | Origin | T | D,S,P | 11672 | -5828.8 | 2 | 0.001 | 1 |
| Model 2 | Weight | T,W | D,S | 11672 | -5828.8 | 2 | 0.09 | 0.75 |
| Model 3 | Length | T,L | D,S | 11671 | -5828.6 | 1 | 0.39 | 0.53 |
| Model 4 | Red spots^a^ | T,R1^a^ | D,S | 11672 | -5828.8 | 2 | 0.08 | 0.77 |
| Model 5 | Redness^b^ | T,R2^b^ | D,S | 11668 | -5827 | 2 | 3.7 | 0.056 |
| Model 6 | Grey value | T,G | D,S | 11672 | -5828.8 | 2 | 0.03 | 0.86 |
| Model 7 | Egg size | T,E | D,S | 11669 | -5827.5 | 1 | 2.8 | 0.09 |
|  |  |  |  |  |  |  |  |  |
| Interaction models: | |  |  |  |  |  |  |  |
| Model 8 | TxP | T | D,S | 11671 | -5828.8 | 1 | 0.008 | 0.9 |
| Model 9 | TxW | T | D,S | 11672 | -5828.8 | 2 | 0.09 | 0.76 |
| Model 10 | TxL | T | D,S | 11671 | -5828.6 | 1 | 0.39 | 0.53 |
| Model 11 | TxR1^a^ | T | D,S | 11670 | -5825.9 | 0 | 5.7 | 0.12 |
| Model 12 | TxR2^b^ | T | D,S | 11672 | -5827 | 2 | 3.6 | 0.3 |
| Model 13 | TxG | T | D,S | 11674 | -5828.2 | 4 | 1.24 | 0.74 |
| Model 14 | TxE | T | D,S | 11662 | -5822.1 | 8 | 14 | 0.003 |

Figure S1: Relationship between hatching time and egg carotenoid content.

(A) Mean hatching times per dam (means of maternal half-sib groups) *versus* carotenoid contents of unfertilized eggs (log_10_-transformed means per dam in µg/ml), and (B) treatment-induced hatching; i.e., mean hatching time per dam in sham-treated controls minus mean hatching time in highest stress treatment (nutrient broth at 1:500) *versus* reduction of carotenoid content from day of fertilization until 14 d after treatment (in µg/ml). Astaxanthin: black symbols; zeaxanthin: grey symbols, lutein: open symbols. See text in main manuscript for statistics. There were no significant effects.

Fig. S1


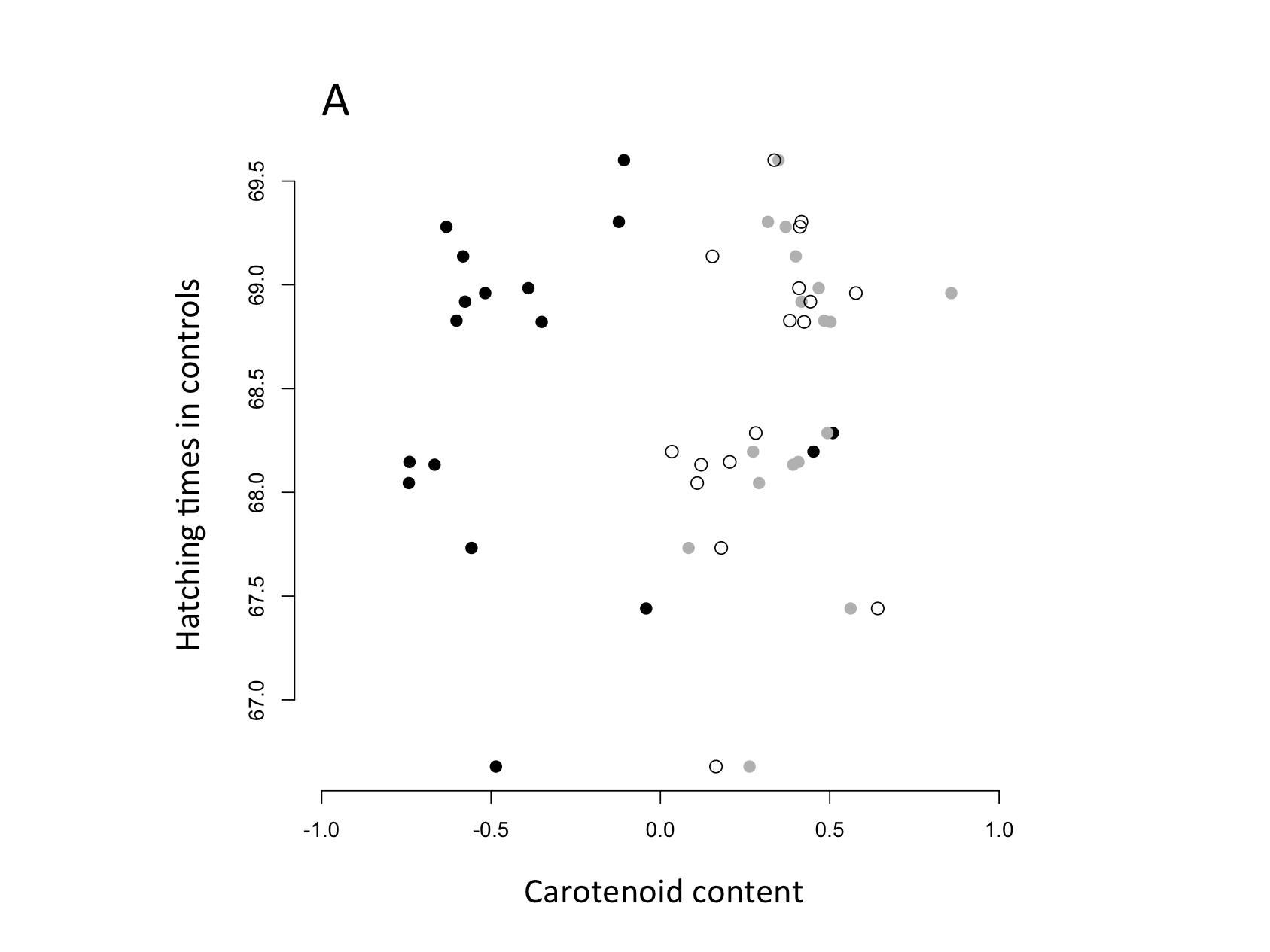


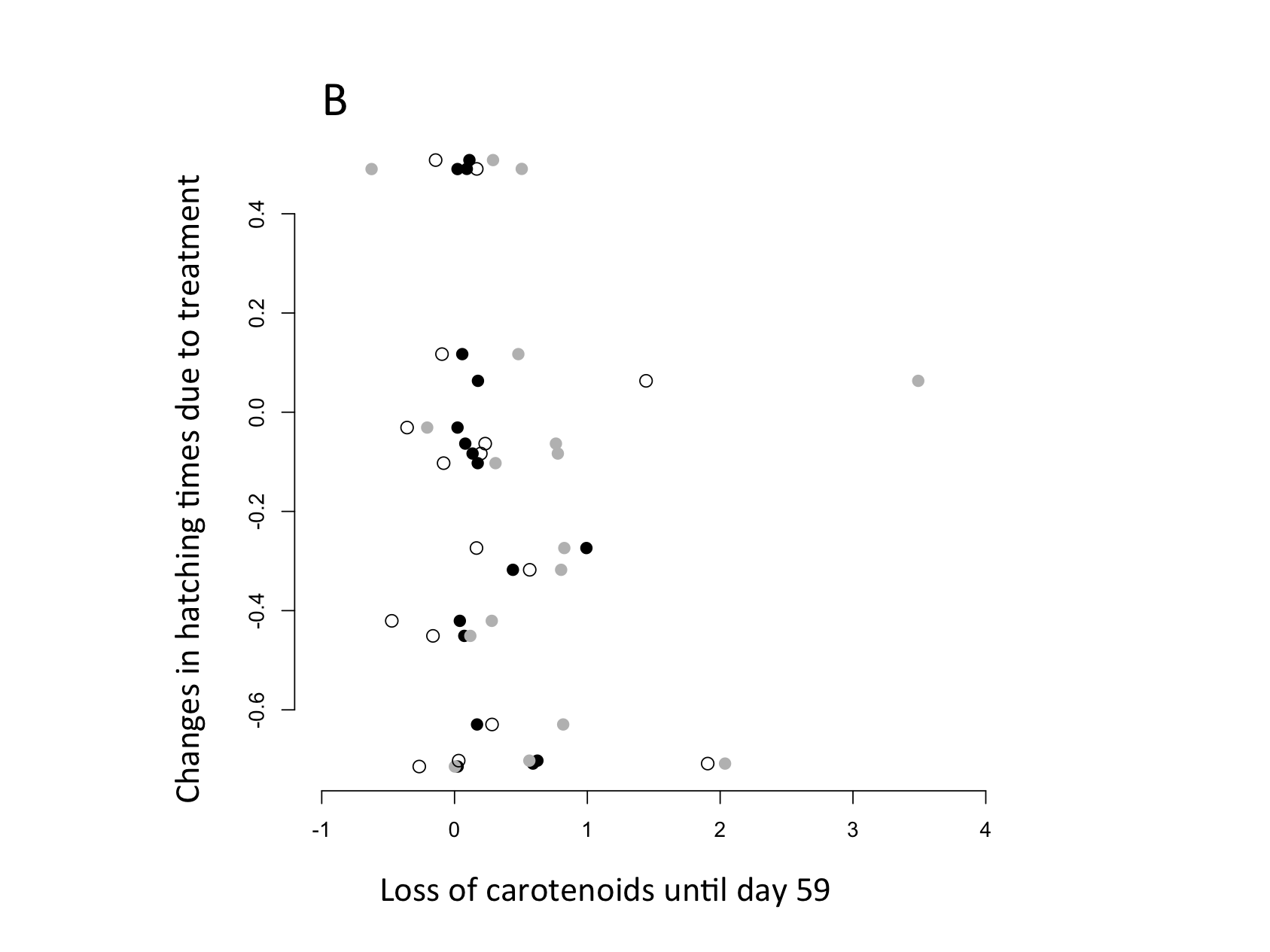

Supplement: Supplementary file 1 [file ECE3-7-5082-s001.docx]
